# Octreotide long-acting release in the treatment of autosomal dominant polycystic kidney disease: a meta-analysis

**DOI:** 10.3389/fendo.2025.1709818

**Published:** 2026-01-21

**Authors:** Mengyan Jin, Ping Shi, Yang Yu

**Affiliations:** 1 Department of Internal Medicine, West China Hospital of Sichuan University, Chengdu, Sichuan, China; 2 Department of Nephrology, People's Hospital of Nanjiang County, Bazhong, Sichuan, China; 3 Department of Nephrology, West China Hospital of Sichuan University, Chengdu, Sichuan, China

**Keywords:** a systemic review, ADPKD, autosomal dominant polycystic kidney disease, clinical effects, octreotide long-acting release

## Abstract

**Objective:**

To systematically evaluate the clinical features and effects of octreotide long-acting release in the treatment of autosomal dominant polycystic kidney disease (ADPKD).

**Methods:**

Databases were searched for randomized controlled trials (RCTs) evaluating octreotide long-acting release (LAR) in ADPKD from inception to December 2024. Two reviewers independently screened studies, extracted data, and assessed the risk of bias. Meta-analysis was performed using Stata 16.

**Results:**

A total of six randomized controlled trials were included. Meta-analysis using a fixed-effects model showed no statistically significant difference in glomerular filtration rate (GFR) between the two groups [SMD = -0.020 (-0.250, 0.210), P = 0.864]. Meta-analysis using a random-effects model showed no statistically significant difference in total kidney volume (TKV) between the two groups [SMD= -0.001(-0.376,0.374), P = 0.996]. A fixed-effects model meta-analysis of a fixed effect model showed a statistically significant reduction in cystic kidney volume (CKV) between the two groups [RR = 1.127 (0.996, 1.276), P = 0.058]. Egger’s test and funnel plot analysis indicated no publication bias for GFR, TKV, or CKV. Sensitivity analysis demonstrated that the study results were robust.

**Conclusions:**

Octreotide long-acting release has a certain effect on reducing cystic kidney volume in patients with ADPKD but shows no significant effect on GFR or TKV and does not increase the risk of adverse reactions. These findings provide a certain reference for the clinical treatment of ADPKD; however, additional high-quality studies are still needed for further validation.

**Systematic Review Registration:**

https://www.crd.york.ac.uk/prospero/, identifier CRD420251242104.

## Introduction

Autosomal dominant polycystic kidney disease (ADPKD) is a single-gene inherited disorder with a global distribution and a considerable incidence, affecting approximately 1 in 1000 individuals (1). With increasing age, numerous cysts of varying sizes develop in both kidneys of ADPKD patients (2). These cysts will continue to enlarge, compress normal renal tissue, and progressively damage kidney structure, ultimately leading to a gradual decline in renal function (3). Most patients eventually develop end-stage renal disease (4, 5) and require kidney replacement therapy to survive. This disease course not only markedly reduces quality of life of patients (6, 7) but also imposes a substantial economic and healthcare burden on families and society. For a long time, the treatment options for ADPKD were relatively limited. Clinical management has mainly focused on controlling blood pressure and managing complications to delay the progression of renal dysfunction; however, these traditional approaches are insufficient to fundamentally halt disease progression (8). In addition to these measures, tolvaptan has also been used in selected ADPKD patients to slow disease progression, although its use is limited by patient eligibility criteria and safety considerations (9).

In recent years, advances in understanding the pathogenesis of ADPKD have significantly expanded insight into this disease. Multiple potential therapeutic targets have been identified at the cellular and molecular levels (10), providing a foundation for the development of novel treatments. Octreotide, as a synthetic somatostatin analog (11), has attracted attention in the field of view of ADPKD treatment due to its unique biological effects, particularly in its long-acting release formulation. Experimental and clinical studies suggest that octreotide LAR may delay the development of ADPKD progression by inhibiting abnormal proliferation of cyst-lining epithelial cells in the lining of renal cysts, reducing excessive synthesis of extracellular matrix synthesis, and modulating relevant signaling pathways (12). However, existing studies evaluating octreotide long-acting release in ADPKD differ in sample size, study design, and outcome measures, leading to inconsistent results and the absence of a clear consensus. The stability of its therapeutic efficacy and the reliability of its safety profile therefore remain uncertain.

In view of this, it is necessary to conduct a systematic study to comprehensively evaluate the clinical value of octreotide long-acting release preparations in the treatment of ADPKD. By conducting a comprehensive literature search and applying rigorous meta-analytic methods, this study aims to synthesize available evidence and provide a more robust, evidence-based reference for the clinical use of octreotide long-acting release preparations in ADPKD management.

## Methods

### Search strategies

This study was conducted as a systematic review and meta-analysis in accordance with the Preferred Reporting Items for Systematic Reviews and Meta-Analyses (PRISMA) guidelines (13) (**Supplementary Table S1**: *PRISMA _2020_checklist*). The meta-analysis was registered on PROSPERO (Registration Number: CRD420251242104). Relevant studies were systematically searched for in the electronic databases PubMed, Web of Science, CNKI, Wan Fang, CQVIP, and Embase. All searches were performed for studies published in English or Chinese up to 15 December 2024. Two authors independently conducted the literature search, and a third author resolved any discrepancies that arose between the two reviewers. Keywords were used for retrieval, and Boolean operators were applied accordingly. The full search strategies for all databases (Web of Science, Embase, CNKI, Wan Fang, and VIP) are provided in the **Supplementary Material** (**Supplementary Table S2**: *Complete Search Strategies for Each Database*).

The PubMed search strategy was as follows:

1. (autosomal dominant polycystic kidney disease) OR (ADPKD) OR (Autosomal Dominant Polycystic Kidney) OR (Adult Polycystic Kidney Disease).
2. (Octreotide Acetate) OR (Sandostatine) OR (Sandostatin) OR (LAR)
3. 1 AND 2

### Inclusion criteria

1. Study design: Randomized controlled trials (RCTs) evaluating octreotide long-acting release in the treatment of ADPKD.
2. Population: Patients diagnosed with autosomal dominant polycystic kidney disease (ADPKD) by clinical or genetic criteria, regardless of age, sex, ethnicity, or disease stage.
3. Intervention: Studies including octreotide long-acting release as the intervention.
4. Comparison: Placebo or other active drugs (e.g., tolvaptan) other than octreotide long-acting release.
5. Outcomes: Primary outcomes included:
  • GFR: Glomerular Filtration Rate(ml/min/1.73 m^2^)
  • TKV: Total Kidney Volume (ml)
  • CKV: Cystic Kidney Volume (ml)
  • Adverse reactions.

#### Exclusion criteria

1. Non-clinical studies: Laboratory or animal-based studies, case reports, reviews, editorials, and letters to the editor.
2. Non-ADPKD populations.
3. Intervention mismatch: Octreotide long-acting release was not used as the intervention.
4. Incomplete data: Studies with incomplete outcome data related to the predefined outcomes.
5. Republication: Duplicate publications or substudies derived from trials already included.

### Data extraction

All retrieved studies were imported into the EndNote X9 software. Duplicate publications and studies with reused data were removed. Two authors independently screened titles and abstracts to identify eligible studies. Full texts of potentially eligible studies were then reviewed to determine if they could be included in the final inclusion. Any disagreements were resolved through discussion or consultation with a third reviewer. Based on the characteristics of the included studies, we extracted the following basic information: first author, year of publication, country, sample size, intervention and control treatments, and reported outcomes.

### Quality of individual studies

The Cochrane Handbook for Systematic Evaluators (5.1.0) (14) was used to assess the methodological quality of the included studies. The assessment included random sequence generation, allocation concealment, blinding, completeness of outcome data, selective reporting, and other potential sources of bias. Each domain was rated as “low risk of bias,” “unclear risk of bias,” or “high risk of bias”.

### Risk of publication bias

Publication bias between studies was assessed using funnel plot symmetry and Egger’s regression test.

### Data analysis

Statistical analyses were performed using Stata 16 software. The χ2 test was used to analyze the heterogeneity among the studies. If *P > 0.1* and *I^2^ < 50%*, statistical heterogeneity among studies was considered low, and a fixed-effects model was used for meta-analysis. If substantial heterogeneity was present, a random-effects model was applied and subgroup analyses were conducted to explore potential sources. If the heterogeneity could not be adequately explained, descriptive analysis was performed. Sensitivity analyses were conducted to assess the robustness of the results. Relative risk (RR) with 95% confidence intervals (CI) was used for dichotomous outcomes, while continuous outcomes were expressed as standardized mean difference (SMD) with 95% confidence intervals. Because SMD cannot be converted into absolute volume units (mL), it was used to reflect the magnitude of the intervention effect. Funnel plots and Egger’s test were used to evaluate publication bias analysis.

## Results

### Selected studies

**Figure 1:**
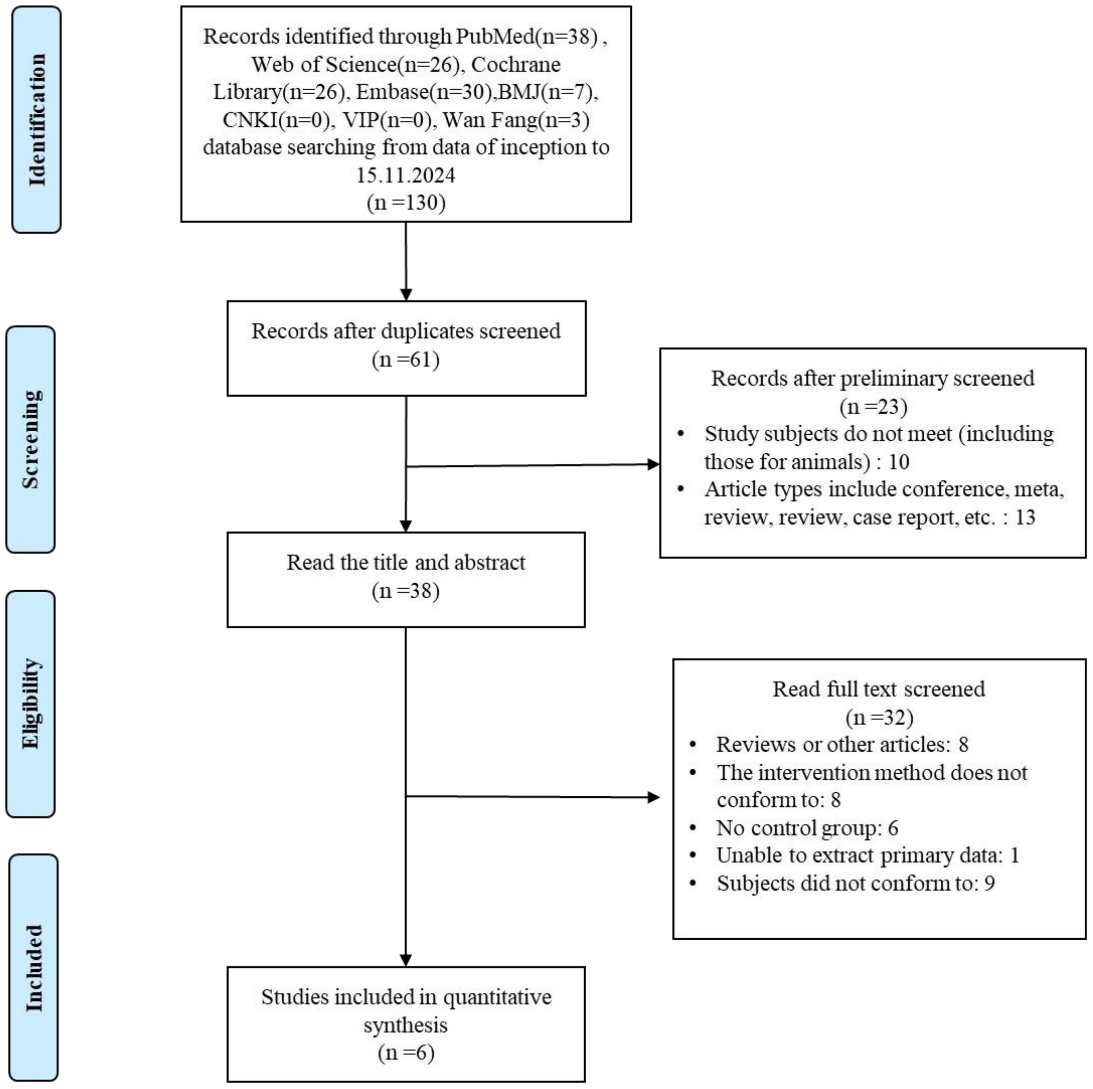
Literature screening process of the meta-analysis.

We followed a step-by-step approach to select eligible studies. All studies identified from the search were exported to EndNote X9 Citation Manager, and we initially excluded 130 articles by title and abstract. 69 duplicate articles were also deleted. After a full evaluation of the remaining articles, 38 articles were excluded. Ultimately, six articles met the inclusion criteria and were included in the meta-analysis. The detailed study selection process is presented in **Figure 1**.

### Study characteristics

The basic characteristics of the included studies are summarized in **Tables 1**, **2**. All six included studies were randomized controlled trials (RCTs). The inclusion and exclusion criteria, as well as the evaluation criteria for each outcome, are detailed in **S3_Supplementary Table 1**.

**Table 1:** Characteristics of included studies.

First Author	Year	Country	Type of operation	Sample	Use of drugs		Sample (Exp/Con/Exit number.)	Treatment cycle(mouth)	GFR measurement methods	Gender (males/Females)		Age (year)	
					Exp.	Con.				Exp.	Con.	Exp.	Con.
Trillini, Matias (15)	2023	Italy	RCT	19	Tolvaptan and Octreotide–Long-Acting Release	Tolvaptan and Placebo	18/19/1	1	1	10/8	10/9	41 ± 12	41 ± 12
Piero Ruggenenti (16)	2005	Italy	RCT	12	Sandostatin-LAR^®^ Depot; Novartis Pharma AG	Placebo	12/12/0	6	1	9.3		44.5 (35–58)	
Norberto Perico (17)	2019	Italy	RCT	100	Octreotide -LAR	Placebo	51/49/30	36	1	31/20	26/23	48.7 ± 8.9	50.0 ± 9.3
Marie C Hogan (18)	2010	USA	RCT	42	Octreotide -LAR	Placebo	28/14/0	12	2	5/23	1/13	49.7 ± 9	50.3 ± 7.3
Marie C Hogan (19)	2012	USA	RCT	34	Octreotide -LAR	Placebo	26/8/0	24	2	NA	NA	NA	NA
Anna Caroli (20)	2013	Italy	RCT	79	Octreotide -LAR	Placebo	40/39/0	36	1	17/23	20/19	36 ± 8	38 ± 8

GFR measurement methods: 1 means the iohexol plasma clearance technique, 2 means the iothalamate clearance technique.

**Table 2:** Primary outcome measure of included studies.

First Author	Year	GFR (ml/min/1.73 m2)		TKV (ml)		CKV (ml)		The number of adverse reaction		The number of Severe adverse reaction rate	
		Exp.	Con.	Exp.	Con.	Exp.	Con.	Exp.	Con.	Exp.	Con.
Trillini, Matias (15)	2023	78 ± 12	79 ± 14	1185 ± 519	1313 ± 625	777 ± 452	883 ± 545	18	17	NA	NA
Piero Ruggenenti (16)	2005	54.0 ± 23.6	57.7 ± 25	2622 ± 1111	2623 ± 1021	1770 ± 941	1762 ± 882	5	2	NA	NA
Norberto Perico (17)	2019	19.63 ± 5.88	21.40 ± 10.73	4282.50 ± 3405.55	3555.60 ± 1803.08	NA	NA	NA	NA	12	11
Marie C Hogan (18)	2010	64.6 ± 25.66	65.7 ± 26.40	1129 ± 796	874 ± 306	NA	NA	NA	NA	NA	NA
Marie C Hogan (19)	2012	57.7 ± 23.5	62.6 ± 29.0	1214 ± 884	885 ± 355	NA	NA	NA	NA	NA	NA
Anna Caroli (20)	2013	76.33 ± 29.47	64.64 ± 41.17	1672.7 ± 1277.56	2621 ± 1713.95	1136.4 ± 1053.6709	1876.5 ± 1381.92	37	*32*	6	*7*

NA indicates unclear or unavailable data. The original data reported by Norberto Perico (2019) were M(IQR); the original data provided by Anna Caroli (2013) were expressed as mean (standard error). Therefore, based on the methods described by Shi et al., these data were recalculated and expressed as mean ± standard deviation.

### Quality assessment

The methodological quality assessment of the included studies is presented in **Table 3**.

**Table 3:** Cochrane risk-of-bias risk assessment tool.

First author	Year	Q1	Q2	Q3	Q4	Q5	Q6	Q7	Q8	Q9	Q10	Q11	Q12	Q13
Trillini, Matias (15)	2023	Yes	Not clear	Yes	Yes	Yes	Not clear	Yes	Yes	Yes	Yes	Yes	Yes	Yes
Piero Ruggenenti (16)	2005	Yes	Not clear	Yes	Yes	Yes	Not clear	Yes	Yes	Yes	Yes	Yes	Yes	Yes
Norberto Perico (17)	2019	Yes	Not clear	Yes	Yes	Yes	Not clear	Yes	Yes	Yes	Yes	Yes	Yes	Yes
Marie C Hogan (18)	2010	Yes	Not clear	Yes	Yes	Yes	Not clear	Yes	Yes	Yes	Yes	Yes	Yes	Yes
Marie C Hogan (19)	2012	Yes	Not clear	Yes	Yes	Yes	Not clear	Yes	Yes	Yes	Yes	Yes	Yes	Yes
Anna Caroli (20)	2013	Yes	Yes	Yes	Yes	Not clear	Yes	Yes	Yes	Yes	Yes	Yes	Yes	Yes

Q1. Whether the random grouping method is really adopted for the research objects; Q2. whether the distribution is hidden; Q3. Whether baseline is comparable between groups; Q4. Whether the blind method was implemented on the research objects; Q5. Whether the intervention was blinded; Q6. Whether the result’s evaluators are blinded; Q7. In addition to the interventions to be verified, whether the other interventions received by the groups were the same; Q8. Whether the follow-up was complete and, if not, whether measures were taken to deal with the loss of follow-up; Q9. Whether all randomly assigned subjects should be included in the result analysis; Q10. Whether the outcome indicators of each group of research objects are assessed in the same way; Q11. Whether the test method of outcome indicators is credible; Q12. Whether the data analysis method is appropriate; Q13. Is the study design reasonable? Are there any differences from standard RCTS in the conduct of research and data analysis.

### Results of index meta-analysis

#### *GFR* (ml/min/1.73 m^2^)

**Figure 2:**
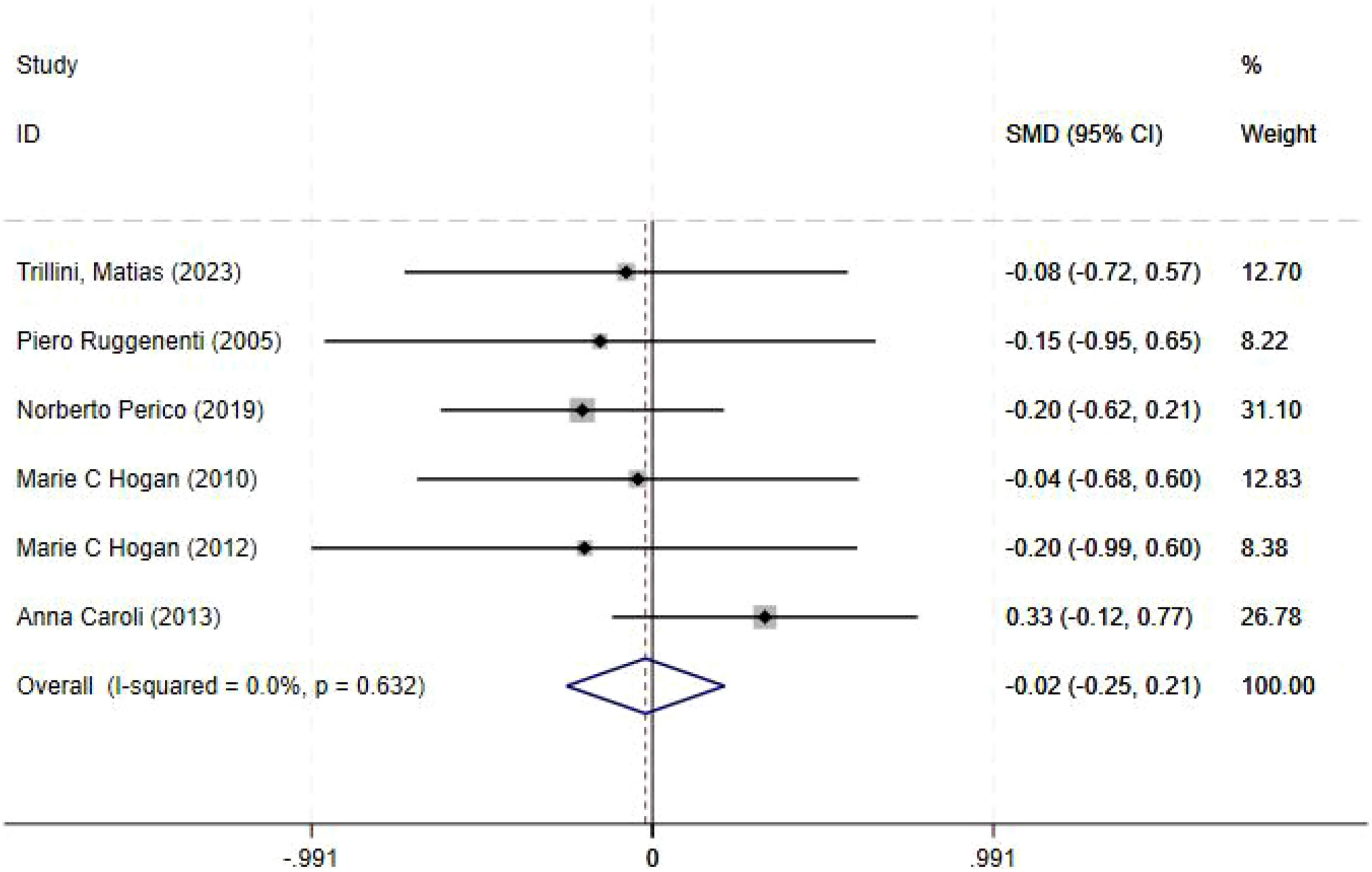
Forest plot comparing GFR (mL/min/1.73 m²) between octreotide long-acting release and control groups.

Glomerular filtration rate was reported in six studies, and the results of an inter-study heterogeneity test were *P =0.632* and *I^2^ = 0.00%*. The meta-analysis using a random-effects model showed that there was no statistically significant difference in the *GFR* between the octreotide long-acting release group and the control group [*SMD= -0.02(-0.25,0.21), P = 0.864*] (**Figure 2**, **Table 4**).

**Table 4:** Effect size.

Index	Heterogeneity test		SMD (95%CI)/ pooled RR (95%CI)	P
	P	I^2^		
GFR(ml/min/1.73m^2^)	0.632	0.00%	-0.020 (-0.250,0.210)	0.864
TKV(ml)	0.046	55.60%	-0.001(-0.376,0.374)	0.996
CKV(ml)	0.349	5.10%	-0.383(-0.731,-0.035)	0.031
Adverse reaction rate	0.546	0.00%	1.127(0.996,1.276)	0.058
Severe adverse reaction rate	0.644	0.00%	1.012(0.569,1.803)	0.966

#### Total kidney volume (ml)

**Figure 3:**
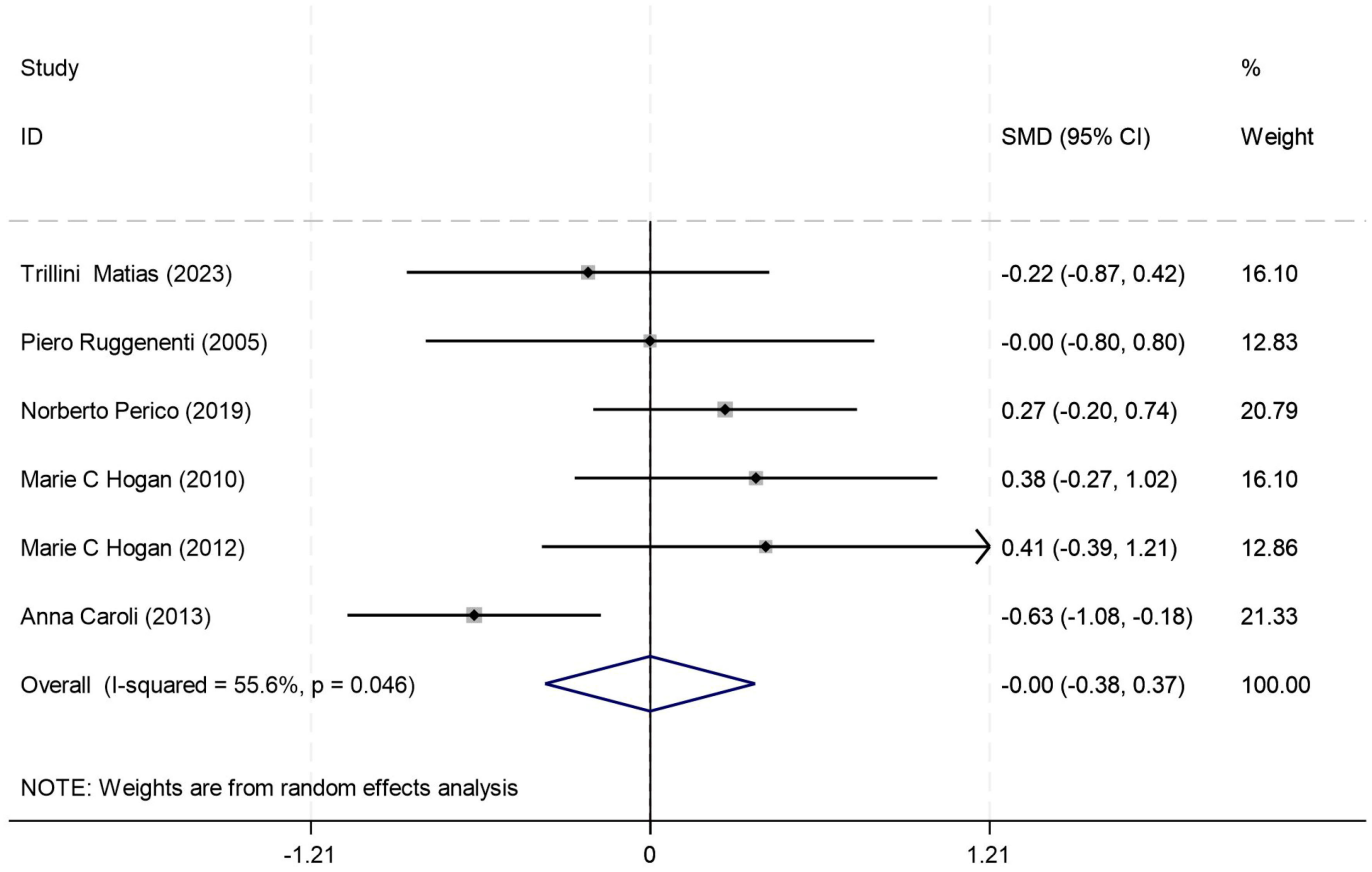
Forest plot comparing TKV (mL) between octreotide long-acting release and control groups.

Total kidney volume was reported in six studies, and the results of an inter-study heterogeneity test were *P = 0.046* and *I^2^ = 55.60%.* The meta-analysis using a random-effects model showed that there was no statistically significant difference in the TKV (ml) between the two groups [*SMD= -0.001(-0.376,0.374), P = 0.006*] (**Figure 3**, **Table 4**).

#### Cystic kidney volume (ml)

**Figure 4:**
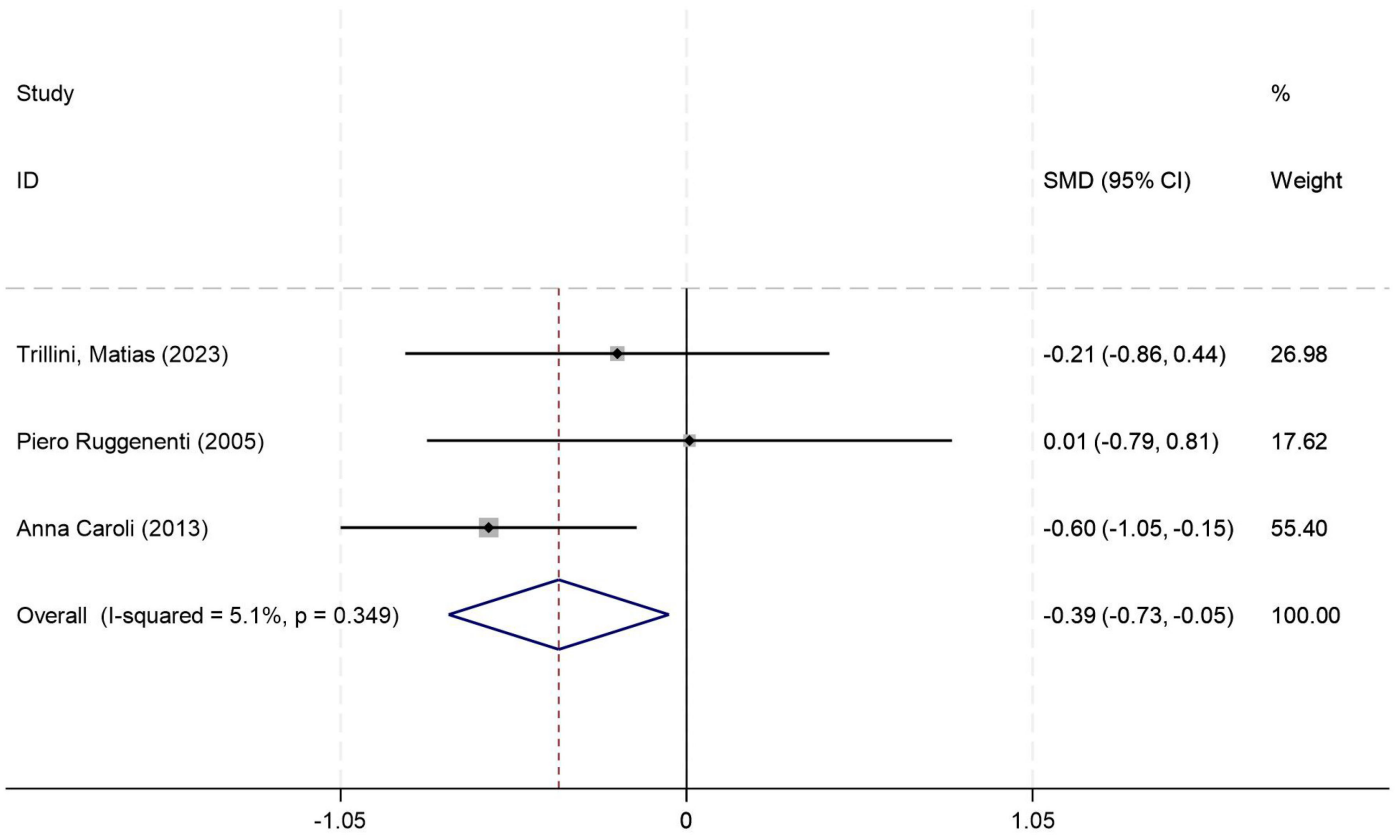
Forest plot comparing CKV (mL) between octreotide long-acting release and control groups.

Cystic kidney volume was reported in six studies, and the results of an inter-study heterogeneity test were *P =0.349* and *I^2^ = 5.10%*. The meta-analysis using a fixed-effects model demonstrated a statistically significant reduction in the *CKV (ml)* between the two groups [*SMD=* -0.383(-0.731,-0.035)*, P = 0.031*] (**Figure 4**, **Table 4**).

### Adverse reaction

**Figure 5:**
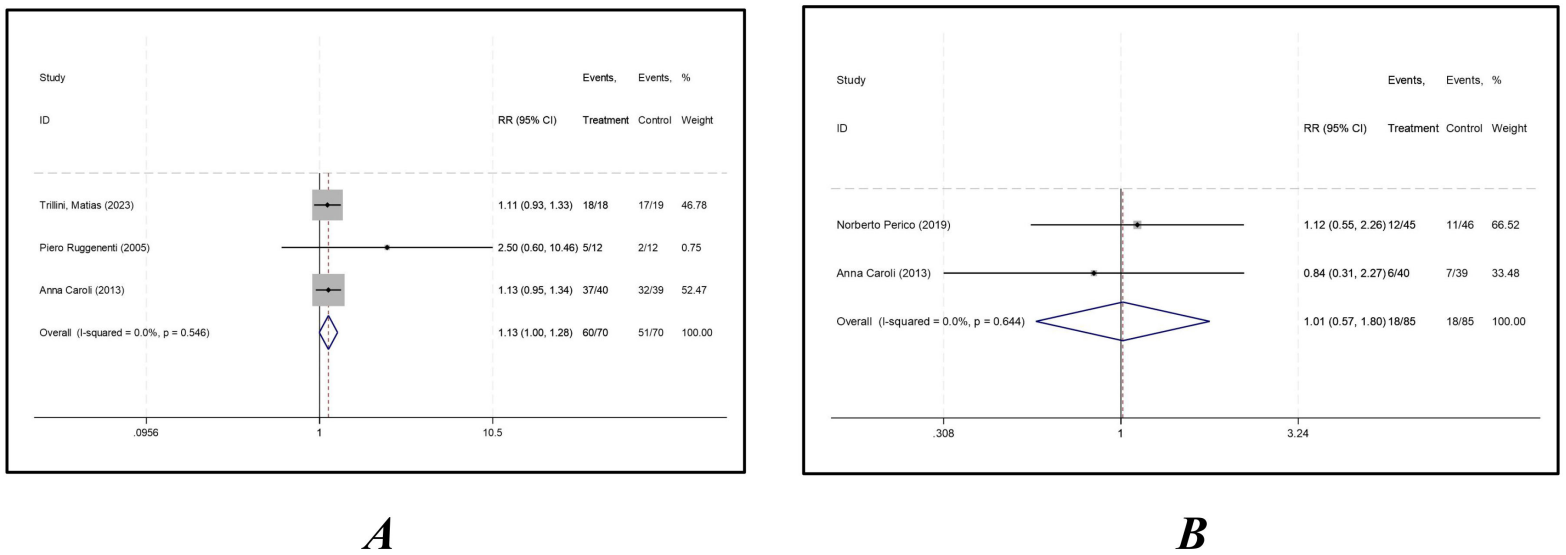
Forest plot comparing adverse reaction rates between octreotide long-acting release and control groups. **(A)** Adverse reaction rate; **(B)** severe adverse reaction rate.

The overall *adverse reaction rate* was reported in three studies, and the results of an inter-study heterogeneity test were *P =0.546* and *I^2^ = 0.00%*. The meta-analysis using a fixed-effects model showed that there was no statistically significant difference in the *adverse reaction* rates between the two groups [*RR = 1.127 (0.996*, *1.276), P = 0.058*] (**Figure 5A**, **Table 2**).

The Severe *adverse reaction* rates were reported in two studies, and the results of an inter-study heterogeneity test were *P = 0.644* and *I^2^ = 0.00%*. The meta-analysis using a fixed-effects model showed that there was no statistically significant difference in severe *adverse reaction* rates between the two groups [*RR = 1.012 (0.569*, *1.803), P= 0.966*] (**Figure 5**, **Table 4**).

### Subgroup analysis

Treatment cycle duration and GFR measurement methods were included in the subgroup analyses. The results showed that neither treatment cycle nor GFR measurement method had a statistically significant effect on GFR, TKV, or CKV (**Table 5**).

**Table 5:** Subgroup analysis of treatment cycle and GRE measurement methods.

Index	Variable	Coef.	Std. Err.	Z	P	95% CI	
GRF	Treatment cycle	0.058	0.161	0.360	0.719	-0.257	0.373
	GRE measurement methods	-0.107	0.317	-0.340	0.736	-0.729	0.515
TKV	Treatment cycle	-0.001	0.013	-0.100	0.923	-0.028	0.025
	GRE measurement methods	0.536	0.421	1.270	0.203	-0.290	1.361
CKV	Treatment cycle	-0.014	0.010	-1.360	0.173	-0.034	0.006
	GRE measurement methods	–	–	–	–	–	

### Publication bias

**Figure 6:**
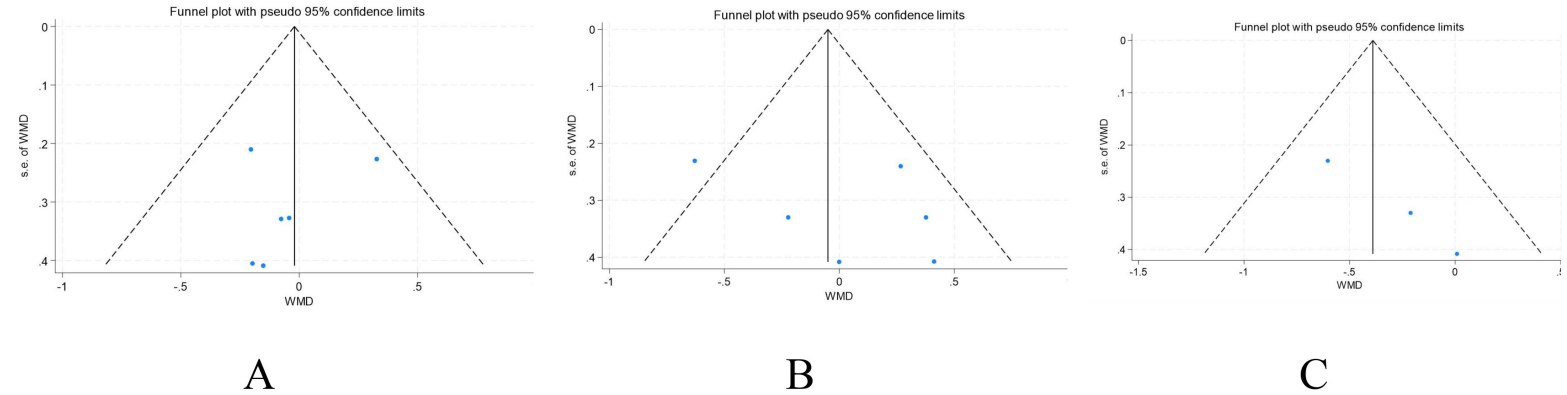
Funnel plots for publication bias assessment. **(A)** GFR(ml/min/1.73m^2^), **(B)** TKV; **(C)** CKV.

Egger’s test results for GFR, TKV, and CKV were *t=-0.61, p = 0.575*; *t=1.00, p = 0.376*; and *t=12.09, p=0.053*, respectively, and the scatter distribution of each study was basically symmetrical, indicating no publication bias (**Figures 6A–C**). Because fewer studies were available for the remaining outcomes, publication bias was not assessed for those indicators.

### Sensitivity analysis

Sensitivity analyses were performed by sequentially excluding each study. The resulting 95% confidence intervals did not cross the null line, and the pooled effect estimates remained within the original 95% confidence intervals, indicating that the results of this study were relatively robust (**Tables 6**–**8**).

**Table 6:** GFR (ml/min/1.73m2) of sensitivity analysis results.

Study omitted	Estimate	95% CI	
Trillini, Matias (15)	-0.012	-0.258	0.234
Piero Ruggenenti (16)	-0.008	-0.248	0.232
Norberto Perico (17)	0.063	-0.214	0.340
Marie C Hogan (18)	-0.017	-0.263	0.229
Marie C Hogan (19)	-0.004	-0.244	0.236
Anna Caroli (20)	-0.147	-0.416	0.122
Combined	-0.020	-0.250	0.210

**Table 7:** TKV (ml) of sensitivity analysis results.

Study omitted	Estimate	95% CI	
Trillini, Matias (15)	0.049	-0.400	0.498
Piero Ruggenenti (16)	0.005	-0.432	0.443
Norberto Perico (17)	-0.067	-0.502	0.368
Marie C Hogan (18)	-0.072	-0.487	0.342
Marie C Hogan (19)	-0.061	-0.471	0.349
Anna Caroli (20)	0.178	-0.105	0.462
Combined	-0.001	-0.376	0.374

**Table 8:** CKV (ml) of sensitivity analysis results.

Study omitted	Estimate	95% CI	
Trillini, Matias (15)	-0.390	-0.962	0.181
Piero Ruggenenti (16)	-0.475	-0.845	-0.105
Anna Caroli (20)	-0.124	-0.627	0.379
Combined	-0.383	-0.731	-0.035

## Discussion

This study comprehensively evaluated the efficacy of octreotide long-acting release in the treatment of ADPKD through a meta-analysis and demonstrated that octreotide long-acting release significantly reduced cystic kidney volume (CKV). From a mechanistic perspective, this finding is consistent with the conclusion of previous basic studies showing that octreotide inhibits abnormal proliferation of epithelial cells in the lining of renal cysts and reduces excessive synthesis of the extracellular matrix (21).

Among the six randomized controlled trials included in this meta-analysis, studies such as that by Anna Caroli et al. (2013) reported CKV data changes in two groups of patients treated with octreotide long-acting release preparation and placebo, providing a key basis for evaluating the effect of octreotide on CKV. Meta-analysis using a fixed-effects model showed a statistically significant difference in CKV between the two groups. However, there were no statistically significant differences in glomerular filtration rate (GFR) or total kidney volume (TKV) between the octreotide long-acting release group and the control group. For GFR, the pooled effect estimate was SMD = 0.048 (95% CI [−0.226, 0.131], P = 0.601), with heterogeneity test results of p = 0.59 and I² = 0.0%. Meta-analysis using a fixed-effects model showed no statistically significant difference in GFR between the two groups. For TKV, the pooled effect estimate was SMD = −0.072 (95% CI [−0.350, 0.205], p = 0.609). Although moderate heterogeneity was present (p = 0.036, I² = 51.4%), random-effects model analysis still showed no statistically significant difference between groups. These findings suggest that the mechanisms underlying ADPKD disease progression are highly complex and involve more than just cyst growth alone (22). In addition to cyst expansion and compression of normal kidney tissue, it also includes renal vascular diseases, such as small arteries in the kidney, which will affect the blood perfusion of the kidney. The inflammatory responses damage renal cells and tissues, while progressive fibrosis further disrupts normal renal structure and function of the kidney (23). Although octreotide can reduce cyst volume to some extent, it may not be able to effectively intervene in these multifactorial pathological processes, which could explain the lack of significant improvement in GFR and TKV.

From a clinical perspective, continuous cyst enlargement is often associated with gradual deterioration of renal function (24). This study demonstrated that CKV can reduce with octreotide treatment, suggesting that, particularly in the early stages of the disease—when cyst-related structural and functional damage is relatively limited—timely use of octreotide long-acting release preparation to control cyst growth may help delay renal function decline. However, although octreotide long-acting release significantly reduced CKV in patients with ADPKD, it did not significantly affect GFR. Whether this CKV reduction ultimately translates into long-term preservation of renal function remains uncertain and requires extended follow-up studies, as the current analysis did not include outcomes such as quality of life or renal replacement therapy. Regarding safety, this meta-analysis found that octreotide long-acting release preparations did not significantly increase the risk of adverse reactions. Although neither overall adverse reactions (RR = 1.127, 95% CI [0.996, 1.276], p = 0.058) nor severe adverse reactions (RR = 1.012, 95% CI [0.569, 1.803], p = 0.966) differed significantly between the octreotide long-acting release and the control groups, the numerical incidence of adverse reactions was slightly higher in the octreotide LAR group. Therefore, clinicians should remain attentive to potential adverse effects when prescribing this therapy.

Recent real-world evidence has been cited to contextualize the clinical value of octreotide long-acting release in ADPKD, particularly in patients with advanced renal dysfunction. Riccio et al. (2024) (25)conducted a single-center retrospective real-life study including 31 patients with ADPKD and stage 4 chronic kidney disease (estimated GFR 15–30 mL/min/1.73 m²) who received regular-dose octreotide long-acting release for at least 2 years. Compared with the pretreatment period (1 year before therapy initiation), the annualized rate of eGFR decline significantly slowed after 1 and 2 years of treatment (p < 0.001). This effect was independent of baseline eGFR, with similar benefits observed in both the 15–24 and 25–30 mL/min/1.73 m² subgroups. In terms of safety, only 19% of patients reported adverse events, primarily biliary tract disorders, and no patients discontinued treatment or required dose reduction due to side effects. These findings are consistent with the safety conclusions of the present meta-analysis, which indicate that octreotide long-acting release does not significantly increase the risk of adverse reactions.

Based on the results of this study, the optimal use of octreotide long-acting release preparation should be further explored. At present, there are differences in the dosage and treatment duration across studies, which may influence therapeutic efficacy. Future high-quality studies are needed to identify the optimal dose and course of octreotide for ADPKD patients at different disease stages and with varying individual characteristics. For example, multicenter, randomized controlled trials could be designed with treatment arms using different octreotide doses and durations, with longitudinal assessment of CKV, GFR, TKV, and other relevant outcomes, as well as the occurrence of adverse reactions, so as to determine the most effective treatment strategy. Given the complexity of the disease mechanism of ADPKD pathogenesis, combination therapy may represent an important direction for future research. Octreotide long-acting release could be evaluated in combination with other treatments (26), such as angiotensin-converting enzyme inhibitors (ACEIs) or angiotensin- II receptor blockers (ARBs), which are widely used to control blood pressure and reduce proteinuria. The renoprotective effects of ACEIs and ARBs in ADPKD have been demonstrated in multiple clinical studies (5, 27, 28). When used in combination with octreotide, these agents may have the potential to intervene in the pathological processes of ADPKD from multiple pathways and more effectively delay disease progression. Previous studies have shown that combination therapy can achieve superior outcomes in other kidney diseases (29–32), providing a reference for exploring octreotide-based combination therapy in ADPKD. To evaluate the combined effects of combination therapy on renal function, cyst volume, and disease progression, large-scale, multi-center, long-term follow-up are needed in the future to track the long-term efficacy of patients using octreotide long-acting release preparations and observe whether new adverse reactions will occur and what long-term effects on kidney function are, such as whether patients can truly reduce the risk of progression to end-stage renal disease. Such evidence would allow a more accurate and comprehensive assessment of the clinical value of this therapy in ADPKD.

## Study limitation

Although six randomized controlled trials were included in this study, the overall sample size of this meta-analysis was relatively small. Limited sample sizes may not adequately capture complex interindividual variability, potentially affecting the accuracy of estimates regarding the efficacy and safety of octreotide long-acting release across diverse patient populations. For example, patients may metabolize and respond differently to the drug, but this study was difficult to analyze in depth due to sample size limitations. In addition, the severity of disease in patients included in the study may be unevenly distributed, which may also affect the generality and reliability of the study results.

## Conclusion

Octreotide long-acting release demonstrates a significant effect in reducing cystic kidney volume in patients with ADPKD but does not show a significant impact on GFR or TKV and does not increase the risk of adverse reactions. These findings provide supportive evidence for the potential clinical use of octreotide long-acting release in ADPKD; however, further high-quality studies are required to confirm its long-term efficacy and safety.

## Data Availability

The raw data supporting the conclusions of this article will be made available by the authors, without undue reservation.
