# Supplementary material for: Octreotide long-acting release in the treatment of autosomal dominant polycystic kidney disease: a meta-analysis
Source: Front Endocrinol (Lausanne). 2026 Jan 21;16:1709818. doi: 10.3389/fendo.2025.1709818 (PMC12870658; doi:10.3389/fendo.2025.1709818)
Supplement: Supplementary file 1 [file Table1.docx]

Supplementary Table 1

| Supplementary Table 1 Inclusion and exclusion criteria | | | | | |
| --- | --- | --- | --- | --- | --- |
| First Author | Year | Experimental group dose | Control group dose | **Inclusion criteria** | **Exclusion criteria** |
| Trillini, Matias | 2023 | Tolvaptan was started at the dose of 45 mg in the morning and 15 mg in the afternoon. Then, morning and afternoon doses were up-titrated every 2 days to 60 and 30 mg and then to 90 and 30 mg, respectively, according to tolerability.According to the randomization plan, at the start of one of the two treatment periods, tolvaptan was combined with two 20-mg i.m. injections of octreotide-LAR | Tolvaptan was started at the dose of 45 mg in the morning and 15 mg in the afternoon. Then, morning and afternoon doses were up-titrated every 2 days to 60 and 30 mg and then to 90 and 30 mg, respectively, according to tolerability. At the start of the other treatment period, tolvaptan was combined with two i.m. injections of 0.9% NaCl solution (placebo for octreotide-LAR) | 1、Adult (>18‐yr‐old) men and women who had signed informed consent and with a clinical and ultrasonographic diagnosis of ADPKD; 2、serum creatinine < 1.2 mg/dl (for man) and < 1.0 mg/dl (for woman) and changes <30% over the last year; 3、 creatinine clearance ≥80 ml/min/1.73m2 measured one to two weeks apart: one during the prescreening or during the screening period (alternatively, two measurements during the screening period, one to two weeks apart) and the other at baseline; 4、female participants must be of non‐childbearing potential or must agree to abstinence or use a highly effective form of contraception. | 1、Pregnant or lactating women and women with childbearing potential who did not agree to abstinence or to use of a highly effective form of contraception were excluded along with patients with concomitant systemic, kidney parenchymal or urinary tract disease; diabetes; 2、urinary protein excretion rate >300 mg/24 hours and/or abnormal urinalysis suggestive of concomitant glomerular disease;  3、symptomatic urinary and/or biliary tract infection or obstruction; 4、hemorrhagic or complicated cysts which might acutely affect kidney function and volumes; QT-related electrocardiography abnormalities; 5、 cancer and major systemic diseases that could prevent completion of the planned follow-up or interfere with data collection or interpretation; 6、 known hypersensitivity to the investigational medical active substances or to any of the excipients or to benzazepine or benzazepine derivatives; 7、elevated liver enzymes and/or signs or symptoms of liver injury prior to initiation of treatment that meet the requirements for permanent discontinuation of Tolvaptan;  8、anuria, volume depletion or hypernatremia;  9、failure to perceive or respond to thirst; 10、ferro-magnetic prosthesis, aneurysm clips, severe claustrophobia or any other contraindication to MRI evaluation; 11、 psychiatric disorders and any condition that could prevent full comprehension of the purposes and risks of the study; |
| Piero Ruggenenti | 2005 | Sandostatin-LAR® Depot; Novartis Pharma AG, Basel, Switzerland:40 mg intramuscularly for 28 days, given as two intragluteal 20 mg injections | placebo: 40 mg intramuscularly for 28 days, given as two intragluteal 20 mg injections | Patients aged 18 years or older, with a clinical and echographic diagnosis of ADPKD and a serum creatinine concentration <3.0 mg/dL, but >1.2 mg/dL (males) or >1.0 mg/dL (females) were selected for study participation. | 1、Patients with concomitant systemic, renal parenchymal or urinary tract disease, diabetes, overt proteinuria (urinary protein excretion rate >1 g/24 hours), or abnormal urinalysis suggestive of concomitant, clinically significant glomerular disease, urinary tract lithiasis, infection or obstruction, biliary tract lithiasis or obstruction, more than two hemorrhagic or complicated cysts, cancer and major systemic diseases that could prevent completion of the planned follow-up or interfere with data collection or interpretation, psychiatric disorders, and any condition that could prevent full comprehension of the purposes and risks of the study were not considered eligible for study participation. 2、Pregnant or lactating women or fertile women without effective contraception were also excluded from the study. |
| Norberto Perico | 2019 | Participants were randomized to receive 2 intramuscular injections of 20 mg octreotide-LAR (n = 51) or 0.9% sodium chloride solution (placebo; n = 49) every 28 days for 3 years | | Adult (>18 years) men and women with ADPKD according to Ravine criteria and eGFR between 15 and 40 ml/min/1.73 m2 were eligible | excluded patients with confounding factors that could affect renal function loss independently of kidney growth and treatment allocation (HbA1c > 8%, systolic/diastolic blood pressure > 180/110 mm Hg, urinary protein excretion > 3 g/24 h); patients with abnormal urinalysis suggestive of concomitant, clinically significant glomerular disease; patients with urinary tract lithiasis or infection; patients with symptomatic gallstones, cancer, or major systemic disease; those who were unable to provide informed consent; and pregnant, lactating, or potentially childbearing women without adequate contraception |
| Marie C Hogan | 2010 | octreotide LAR depot (up to 40 mg every 28 ± 5 days) or placebo for 1 year | | \ | 1、Unable to ravel to Mayo Cinic 2、Liver disoase too mild 3、Recent iver surgery 4、Other pahology considered significart |
| Marie C Hogan | 2012 | Up to 40 mg, once every 28+/-5 days | Up to 40 mg, once every 28+/-5 days | 1、 Men and women ≥18 years with a diagnosis of ADPKD (meeting Ravine’s criteria) or ADPLD (defined by the criteria described by Reynolds et al.) 2、severe PLD defined as a liver volume >4000 mL or symptomatic disease due to mass effects from hepatic cysts 3、who were not candi_x005fdates or declining surgical intervention were eligible  4、 Patients had to be willing to travel to Mayo Clinic, Rochester, MN. | 1、inability to provide informed consent, women of childbearing potential unwilling to employ adequate contraception 2、serum creatinine concentration >3 mg/dL or dialysis dependency, symp_x005ftomatic gallstones or biliary sludge, uncontrolled hypertension (systolic blood pressure >160 mmHg and diastolic blood pressure >100 mmHg) or diabetes mellitus, cancer 3、major systemic diseases that could prevent completion of the planned follow-up or interfere with data collection  4、 interpretation and current or prior use of somatostatin analog within 6 months of enrollment or history of significant adverse reaction from a somatostatin analog |
| Anna Caroli | 2013 | Up to 40 mg, once every 28+/-5 days | Up to 40 mg, once every 28+/-5 days | Adult (>18 years of age) men and women with an estimated glomerular filtration rate (GFR) of 40 mL/min/1·73 m2 or higher clinically and ultrasonically diagnosed with autosomal dominant polycystic kidney disease according to Ravine criteria, calculated according to the four variable equations of the Kidney Disease Diet Adjustment Study, were eligible. | 1. Patients with confounding factors that may affect the loss of kidney function, unrelated to kidney growth and treatment allocation, i.e., diabetes; Urinary protein excretion rate was greater than 1 g/24 h; 2. Abnormal urine analysis suggests concomitant, clinically significant glomerular disease; 3. Urinary calculi or infection. Symptomatic gallstones or biliary mud, cancer; 4. Patients with major systemic diseases, patients who are unable to provide informed consent, and women who are pregnant, breastfeeding or at risk of giving birth who are not using appropriate contraception are also excluded." |
