# Supplementary material for: Octreotide long-acting release in the treatment of autosomal dominant polycystic kidney disease: a meta-analysis
Source: Front Endocrinol (Lausanne). 2026 Jan 21;16:1709818. doi: 10.3389/fendo.2025.1709818 (PMC12870658; doi:10.3389/fendo.2025.1709818)
Supplement: Supplementary file 2 [file Table2.pdf]

Table Search Strategies for Each Database

Database	Search Strategies	Search results
Pubmed	1：(((autosomal dominant polycystic kidney disease) OR (ADPKD)) OR (Autosomal Dominant Polycystic Kidney)) OR (Adult Polycystic Kidney Disease)2：(((Octreotide Acetate) OR (Sandostatine)) OR (Sandostatin)) OR (LAR)3：(#1) AND (#2)	38
web of sicience	(TS=((((autosomal dominant polycystic kidney disease) OR (ADPKD)) OR (Autosomal Dominant Polycystic Kidney)) OR (Adult Polycystic Kidney Disease))) AND TS=((((Octreotide Acetate) OR (Sandostatine)) OR (Sandostatin)) OR (LAR)) and Preprint Citation Index (Exclude – Database)	26
Cochrane Library	1：(((autosomal dominant polycystic kidney disease) OR (ADPKD)) OR (Autosomal Dominant Polycystic Kidney)) OR (Adult Polycystic Kidney Disease)2：(((Octreotide Acetate) OR (Sandostatine)) OR (Sandostatin)) OR (LAR)3：(#1) AND (#2	26
embase	(autosomal AND dominant AND polycystic AND kidney AND disease OR (autosomal AND dominant AND polycystic AND kidney) OR (adult AND polycystic AND kidney AND disease) OR adpkd) AND (octreotide AND acetate OR sandostatine OR sandostatin OR lar)	30
BMJ	for abstract or title "(autosomal dominant polycystic kidney disease) OR (ADPKD) OR (Autosomal Dominant Polycystic Kidney) OR (Adult Polycystic Kidney" (match all words) and full text or abstract or title "(Octreotide Acetate) OR (Sandostatine) OR (Sandostatin) OR (LAR)" (match whole all)	7
CNKI	(FT='常染色体显性遗传多囊肾病' OR FT='常染色体显性遗传性多囊肾病' OR FT='成人型多囊肾' OR FT='常染色体显性多囊肾') AND(FT='醋酸奥曲肽' OR FT='善龙' OR FT='善宁')	0
Wanfang	(全部:(醋酸奥曲肽) or 全部:(善龙) or 全部:(善宁)) and (全部:(常染色体显性遗传多囊肾病；) or 全部:(成人型多囊肾；) or 全部:(常染色体显性遗传性多囊肾病；) or 全部:(常染色体显性多囊肾))	3
Vip	(U='常染色体显性遗传多囊肾病' OR U='常染色体显性遗传性多囊肾病' OR U='成人型多囊肾' OR U='常染色体显性多囊肾') AND(U='醋酸奥曲肽' OR U='善龙' OR U='善宁')	0
